# Protocol for obtaining cancer type and subtype predictions using subSCOPE

**DOI:** 10.1016/j.xpro.2025.103705

**Published:** 2025-04-10

**Authors:** Jasleen K. Grewal, A. Gordon Robertson, Kyle Ellrott, Christopher K. Wong, Jordan A. Lee, Christina Yau, Bahar Tercan, Mauro A.A. Castro, Christopher C. Benz, The Cancer Genome Atlas Analysis Network, Theo A. Knijnenburg, Mauro A.A. Castro, Vinicius S. Chagas, Victor H. Apolonio, Verena Friedl, Joshua M. Stuart, Vladislav Uzunangelov, Christopher K. Wong, Jesper B. Andersen, Andrew D. Cherniack, Galen F. Gao, Gad Getz, Stephanie H. Hoyt, Whijae Roh, Lindsay Westlake, Christopher C. Benz, Jasleen K. Grewal, Steven J.M. Jones, A. Gordon Robertson, Samantha J. Caesar-Johnson, John A. Demchok, Ina Felau, Anab Kemal, Roy Tarnuzzer, Zhining Wang, Liming Yang, Jean C. Zenklusen, Rehan Akbani, Bradley M. Broom, Zhenlin Ju, Andre Schultz, Akinyemi I. Ojesina, Katherine A. Hoadley, Avantika Lal, Daniele Ramazzotti, Chen Wang, Alexander J. Lazar, Lewis R. Roberts, Taek-Kyun Kim, Ilya Shmulevich, Bahar Tercan, Paulos Charonyktakis, Vincenzo Lagani, Ioannis Tsamardinos, Esther Drill, Ronglai Shen, Martin L. Ferguson, Kami E. Chiotti, Kyle Ellrott, Brian J. Karlberg, Jordan A. Lee, Eve Lowenstein, Adam Struck, Paul T. Spellman, Christina Yau, Toshinori Hinoue, Peter W. Laird, Jean C. Zenklusen, Andrew D. Cherniack, Peter W. Laird, Steven J.M. Jones

**Affiliations:** 1 Canada’s Michael Smith Genome Sciences Centre, BC Cancer, Vancouver, BC V5Z 4S6, Canada; 2 Oregon Health and Science University, Portland, OR 97239, USA; 3 UC Santa Cruz Genomics Institute and Department of Biomolecular Engineering, Santa Cruz, CA 95060, USA; 4 University of California, San Francisco, Department of Surgery, San Francisco, CA 94158, USA; 5 Buck Institute for Research on Aging, Novato, CA 94945, USA; 6 Institute of Systems Biology, 401 Terry Avenue North, Seattle, WA 98109, USA; 7 Bioinformatics and Systems Biology Laboratory, Federal University of Paraná, Curitiba, Paraná 81520-260, Brazil; 8 Center for Cancer Genomics, National Cancer Institute, Bethesda, MD 20892, USA; 9 The Broad Institute of Harvard and MIT, Cambridge, MA 02142, USA; 10 Department of Medical Oncology, Dana-Farber Cancer Institute, Boston, MA 02215, USA; 11 Harvard Medical School, Boston, MA 02115, USA; 12 Department of Epigenetics, Van Andel Institute, Grand Rapids, MI 49503, USA

**Keywords:** Bioinformatics, Cancer, Genomics, Computer sciences

## Abstract

We present a protocol for obtaining cancer type and subtype predictions using a machine learning method (subSCOPE). We describe steps for data preparation, subSCOPE setup, and running subSCOPE inference on prepared data. The protocol supports five -omics data types as input (DNA methylation, gene expression, microRNA [miRNA] expression, point mutations, and copy-number variants) and allows individual cancer type and data type selection. For non-The Cancer Genome Atlas (TCGA) cancer samples, it provides subtype-level classification across 26 different TCGA cancer cohorts and 106 subtypes.

For complete details on the use and execution of this protocol, please refer to Ellrott et al.^1^

## Highlights

• Classify -omics data into one of 106 subtypes across 26 human cancers with subSCOPE
• Use gene expression, miRNA, mutation, copy-number variation, or methylation data
• Specify choice of data types and cancer types for prediction if desired
• Obtain confidence values associated with each prediction

## Abstract (editor highlights)

Publisher’s note: Undertaking any experimental protocol requires adherence to local institutional guidelines for laboratory safety and ethics.

## Graphical abstract

**Figure:**
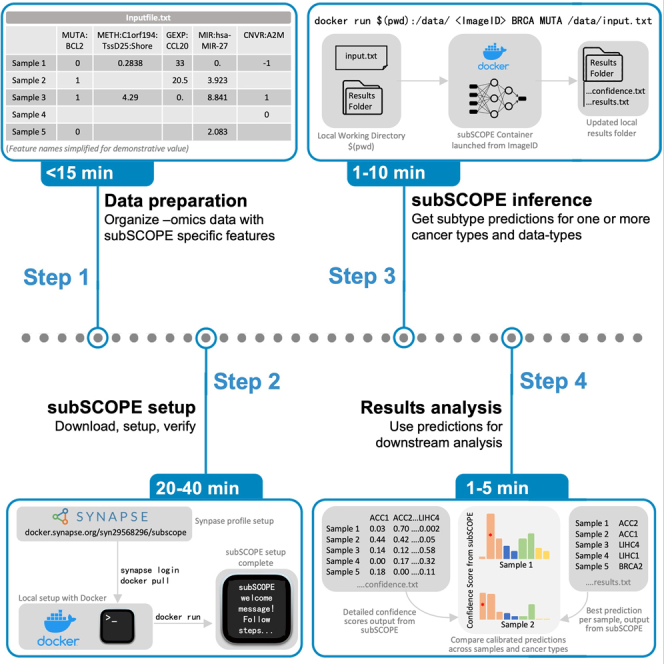

## Before you begin

### Background on automated cancer subtyping from -omics data

Cancer diagnosis using gene-based features can be recast as either a cancer-specific or a subtype-specific classification problem. Our previous work has shown the utility of neural network-based pan-cancer classification using transcriptomic data.^2^ We propose subSCOPE, a machine-learning (ML) framework that extends a previously published ML model called SCOPE^2^ to simultaneously classify new cancer samples into a cancer type and subtype. SubSCOPE also expands the data-types supported beyond bulk RNA-seq to include miRNA expression, DNA methylation, point mutations, and copy number variants.^1^ For further information on the cancer and subtype definitions used within each data-type, the reader is referred to the companion paper, which consolidates the training data.^1^

The protocol shared here describes the specific steps for obtaining cancer type and subtype predictions using a version of subSCOPE that has already been trained on TCGA cancer type and subtype data, and can be utilized for non-TCGA cancer samples. We provide optional recipes for subSCOPE model training as Document S1*: Training Guide and Summary*.

### Institutional permissions

To train the subSCOPE ML model version provided in this protocol, we used the Cancer Genome Atlas (TCGA) Research Network tumor and matched normal samples with informed consent under their local Institutional Review Boards. The model was trained as part of research requirements in the Tumor Molecular Pathology (TMP) Analysis Working Group of the Genomic Data Analysis Network (GDAN).

### Hardware

Local memory: a minimum of 16 GB recommended.

Local storage: a minimum of 4.5 GB required.

Use a CPU for making predictions with this model; a GPU is not required.

***Note:*** To replicate the model training (optional) described in Document S1*: Training Guide and Summary*, use hardware equivalent or better than an NVIDIA V100 GPU with 32 GB of memory.

### Download and set up the subSCOPE Docker container

**Timing: <45 min**

1. Set up python3 by downloading it from https://www.python.org/downloads.
2. Verify that you have python3 correctly configured by running the following command:

> python3 –version

The following output will appear, where *x* will differ depending on the sub-version of Python that is downloaded.

Python 3.x.x

***Note:*** The steps presented here are tested with Python 3.8.5.

3. Set up Synapse.
  a. Login or create an account with Synapse at https://www.synapse.org/#!RegisterAccount:0.
  b. Download the Synapse client from https://help.synapse.org/docs/Installing-Synapse-API-Clients.1985249668.html.
    ***Note:*** The steps presented here are tested with Synapse Client 2.4.0.
  c. Login to Synapse by running the following command in the local terminal and enter the synapse account credentials when prompted:
    > synapse login --remember-me
  d. Once successfully logged in to Synapse, the following output will appear:
    Welcome, !
    Logged in as: <your Synapse username>
4. Download and launch Docker^3^ locally by following the instructions at https://www.docker.com/products/docker-desktop/.
  ***Note:*** The steps presented here are tested with Docker version 20.10.14, build a224086.
  a. After completing installation, log in on Docker Desktop with your Docker ID. For this step, use your Docker Username and password. From the terminal, the login command will be.
    > docker login
  b. The output on your screen after a successful login will be:
    Login Succeeded
    Logging in with your password grants your terminal complete access to your account.
  c. From the terminal, log in to the Synapse Docker Registry to access the subSCOPE Docker image from where it is stored on Synapse. For this step, use the Synapse username and password from your Synapse account.
    > docker login -u <your Synapse username> docker.synapse.org
  d. The output on your screen after a successful login will be:
    Login Succeeded
5. Obtain the pre-trained subSCOPE Docker image from https://www.synapse.org/#!Synapse:syn30986019.
  ***Note:*** The subSCOPE pre-built image is 2.5 GB. This is the most time-consuming setup step depending on the user’s available internet bandwidth.
  a. From the terminal or command-line, navigate to the project root directory.
  b. Complete the three necessary logins described in the previous steps. They are summarized here again for reference:
    > synapse login –-remember-me
    > docker login
    > docker login -u <username> docker.synapse.org
  c. Copy the subSCOPE Docker container from the Synapse link using the command:
    > docker pull < docker.synapse.org/syn29568296/subscope
    Figure 1 shows example logs when the download is complete.
    ***Note:*** Alternatively, download the Docker image manually and build it using the following two commands:
    > synapse get syn30986019

**Figure 1:**
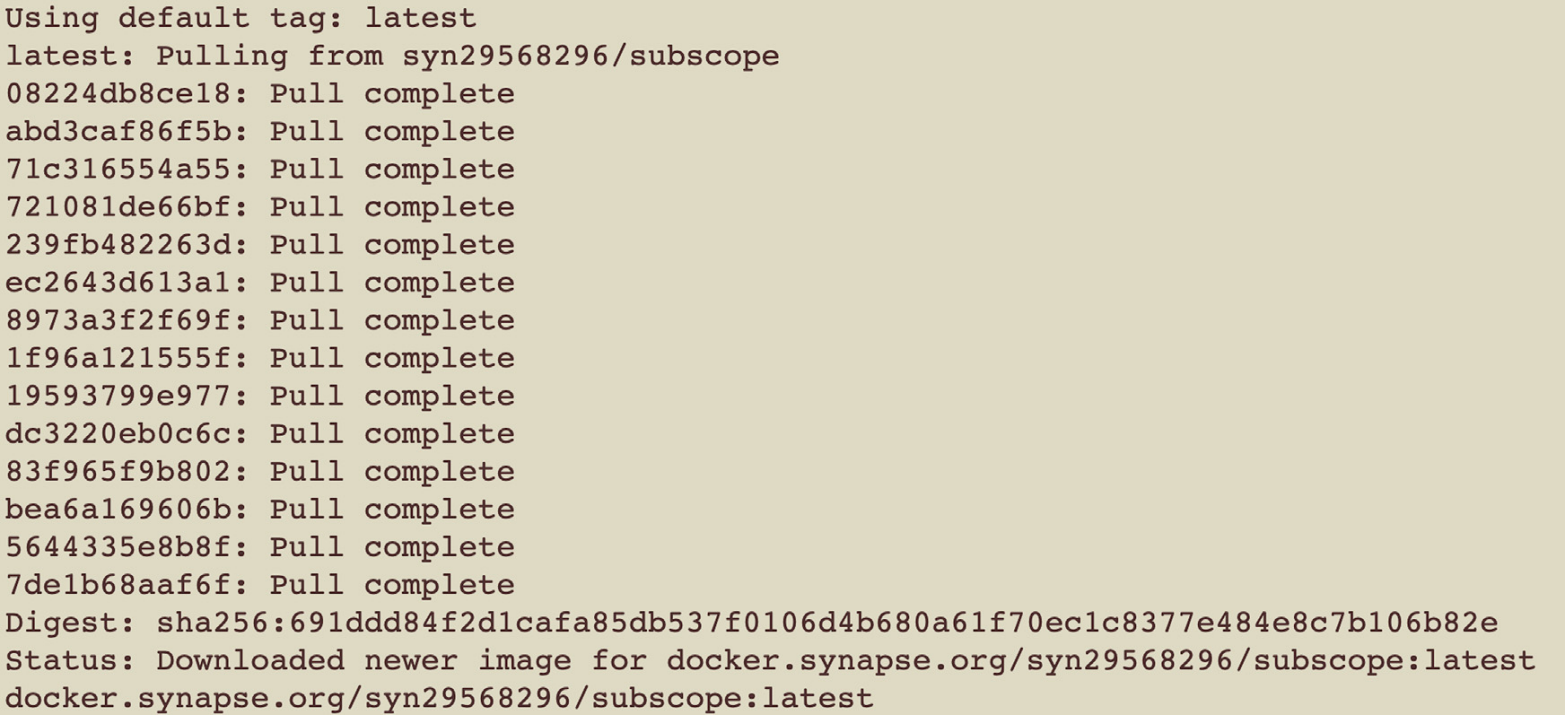
Expected output from downloading subSCOPE Docker container from Synapse
    Load the Docker image from this .tar.gz file and create a container.
    > docker load --input dockerimage-subscope-ccg-tmp.tar.gz
6. Confirm that the Docker image has been loaded by running the following command:

> docker images

Look for an entry under the column ‘REPOSITORY’ that says

‘docker.synapse.org/syn29568296/subscope’

The IMAGE ID for each entry will differ for each downloaded instance. Use this Image ID in the next step.

7. Launch a Docker Container with this image to understand the necessary inputs.

> docker run -t <IMAGE ID>

Or alternatively,

> docker run subscope

**Figure 2:**
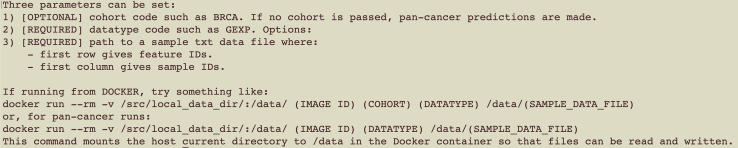
Expected output from subSCOPE Docker container on successful setup

A screenshot of the expected output is shown in Figure 2.

***Note:*** This will output a guide of the necessary inputs and how to format them.

8. For the implementation of comparator cancer classification models from *Ellrott et al*.,^1^ follow the setup instructions from the NCI GDAN project GitHub repository at https://github.com/NCICCGPO/gdan-tmp-models/blob/main/README.md.
  a. Download the GitHub repository with additional tools and pre-processing scripts from https://github.com/NCICCGPO/gdan-tmp-models.
  b. Navigate to the indicated link and click on the green ‘Code’ tab. Download the ZIP folder by clicking on ‘Download ZIP’. This contains various tools, including subSCOPE, some example data, a tutorial in .md format, and a ReadMe file.
  c. After downloading the ZIP folder, extract all files locally.

### Prepare input files

9. Format the input files correctly.
  a. Format the input as one sample in each row. The first row contains the feature IDs.
    ***Note:*** In the work reported here, the following codes indicate the five main data-types: CNVR (copy number variants), GEXP (gene expression by RNA-seq), METH (DNA methylation), MIR (miRNA mature strand expression), and MUTA (somatic mutations).
    ***Note:*** Feature IDs are named following the nomenclature defined by Ellrott et al.^1^ Feature lists for each data-type (CNVR, GEXP, METH, MIR, MUTA) are also included in supplemental information*‘*Data S1’.
  b. Format the input as one feature in each column. The first column contains the sample IDs.
10. Ensure the values for each data-type are specified correctly.
  a. For GEXP and MIR data, input RPKM or TPM values. Do not log transform the data, as subSCOPE will do this automatically.
  b. For METH data, input raw numeric values. Do not log transform the data, as subSCOPE will do this automatically.
  c. For MUTA data, input discrete, positive integer values.
  d. For CNVR data, input −1 for deletion, 0 for neutral, and 1 for gain.

**Figure 3:**
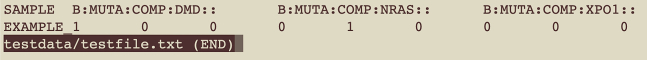
Example input file for subSCOPE

***Note:*** An example input file is shown in Figure 3. An example input file is also included in supplemental information as ‘Data S2’.

## Key resources table

**Table:** 

REAGENT or RESOURCE	SOURCE	IDENTIFIER
**Deposited data**		

subscope.tar.gz (Docker image of pre-trained subSCOPE)	This article	syn30986019
Model training data	Ellrott et al.^1^	TMP_v12_20210228.tar.gz https://gdc.cancer.gov/about-data/publications/CCG-TMP-2022
Supplementary files on Mendeley Data	This article	https://doi.org/10.17632/vd6tct9xwr.1

**Software and algorithms**		

gdan-tmp-models	Ellrott et al.^1^	https://github.com/NCICCGPO/TMP
Synapse client (version 2.4.0)	N/A	https://help.synapse.org/docs/Installing-Synapse-API-Clients.1985249668.html
Synapse account	N/A	https://help.synapse.org/docs/Managing-Your-Account.2055405596.html
Python3 (version 3.8.5)	N/A	https://www.python.org/downloads/
Docker (version 20.10.14)	N/A	https://www.docker.com

**Other**		

Hardware for inference	This article	MacOS v10.15.7, 2.5 GHz Quad-core intel core i7 processor, 16 GB memory

## Materials and equipment

The pre-trained subSCOPE model described in the main protocol is trained using Model Training Data noted in key resources table and following the two general recipes in Document S1*: Training Guide and Summary***.**

## Step-by-step method details

Follow these steps to use the accompanying pre-trained subSCOPE tool and make cancer subtype predictions for new samples. Before beginning, follow instructions in the ‘before you begin’ section to set up the input data file and appropriate software correctly.

**CRITICAL:** For each data-type, subSCOPE determines the cancer type and subtype for a new cancer sample from across 26 cancer types and 106 subtypes - with the exceptions of the MIR data-type for LGGGBM, LIHCCHOL and KIRCKICH due to library construction protocol inconsistencies.

### Data pre-processing

**Timing: <15 min**

1. Create a tab-separated input file with unique sample names in the first column and feature names in the first row.

***Note:*** Ensure feature names correspond to the GDAN TMP specific feature IDs^1^ for each data-type. If needed, use *tools/convert.py* in the GitHub repository to convert them. Feature lists for each data-type (CNVR, GEXP, METH, MIR, MUTA) are also included in supplemental information*‘*Data S1*’.*

***Note:*** The first column is read in as the index column. The first row is read in as the header row.

***Note:*** Use the INPUTFILE from this step as input for running subSCOPE.

2. For CNVR data, use only values [-1, 0, 1], representing somatic copy number loss (all gene level deletions), neutral, and gain (all gene level amplifications) respectively.
3. For GEXP data, quantile-rescale the data but do not log transform it. If needed, use *tools/run_transform.sh* in the GitHub repository to perform this rescaling.
4. For METH and MIR data, do not log transform the input measurements.
5. For MUTA data, use only values [0, 1], representing the absence or presence of a somatic point mutation at the relevant gene coordinate indicated in the feature name.

***Note:*** Transform the input data automatically using subSCOPE: Clip continuous data measurements with negative values (GEXP, MIR) at 0. Log2-transform all continuous data-types (GEXP, MIR, METH) after adding ‘1’ to prevent infinite values.

***Note:*** Transform the input data automatically using subSCOPE: One-hot encode discrete data measurements (CNVR, MUTA). Map a set of categories to a binary encoded matrix. For example, encode Category 1 as {1 0 0}, Category 2 as {0 1 0}, Category 3 as {0 0 1}.

**CRITICAL:** In the current pretrained version of subSCOPE, prior to training no other data filtering, pre-processing, or feature selection is done. The default preprocessing steps of filtering for appropriate feature names and quantile normalization are described further at https://github.com/NCICCGPO/gdan-tmp-models/tree/main/tutorial.

**CRITICAL:** If the input dataset is missing any of the features, missing values will automatically be replaced by 0.

**CRITICAL:** If input dataset has more features than the set required for the specified data-type (CNVR, GEXP, METH, MIR, or MUTA), subSCOPE will automatically subset the input dataset to the required set of features.

### Run subSCOPE

**Timing: <10 min**

***Note:*** Timing assumes a minimum 16 GB RAM and an i7 CPU or equivalent processor.

6. Follow the ‘download and set up the subSCOPE docker container’ sub-section in the ‘before you begin’ section to obtain the Image ID for the subSCOPE Docker container.
7. Run subSCOPE with the active docker container and INPUTFILE:

**Figure 4:**
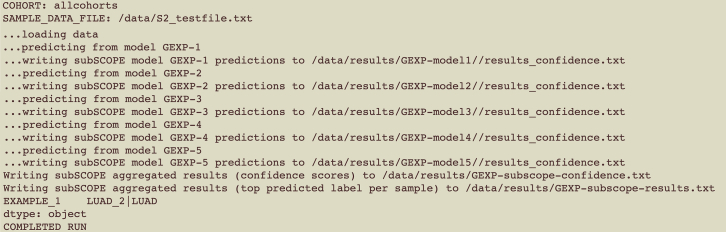
Example logs from subSCOPE for a successful run

> docker run --rm -v $(pwd)/:/data/ <Image ID> <TCGA CODE> <DATA TYPE> /data/<INPUTFILE>

***Note:*** Use the TCGA CODE and DATA TYPE options enumerated at the GitHub Readme for subSCOPE to choose the TCGA CODE and DATA TYPE for the prediction. The following codes are options for the TCGA CODE: allcohorts, ACC, BLCA, BRCA, CESC, COADREAD, ESCC, GEA, HNSC, KIRCHKICH, KIRP, LGGGBM, LIHCCHOL, LUAD, LUSC, MESO, OV, PAAD, PCPG, PRAD, SARC, SKCM, TGCT, THCA, THYM, UCEC, UVM. The following codes are options for the DATA TYPE: CNVR, GEXP, METH, MIR, MUTA.

***Note:*** A successful execution will result in a series of updates logged to the screen, along with the output file. An example of expected output is shown in Figure 4.

8. Follow this example to run subSCOPE and get predictions for the breast cancer (BRCA) subtypes, using gene expression (GEXP) data as the INPUTFILE.

> docker run --rm -v $(pwd):/data/ <Image ID> BRCA GEXP /data/<INPUTFILE>

***Note:*** Replace the code ‘BRCA’ with ‘allcohorts’ to obtain pan-cancer predictions instead of predictions within BRCA subtypes only.

9. Follow this example to run subSCOPE and get predictions across all 26 cancer types and 106 subtypes (‘allcohorts’) using mutation data (‘MUTA’).

> docker run --rm -v $(pwd):/data/ subscope allcohorts MUTA /data/<INPUTFILE>

***Note:*** Refer to the TCGA codes and DATA TYPE codes listed in the preceding step 2 for other options that would be accepted.

10. Retrieve results from the current working directory in a new subfolder called ‘results’.
  a. Use the *[data-type]-subscope-results.txt* file to get the subtype label predicted with highest confidence for each sample.
    ***Note:*** The first column contains sample IDs matching the first column of the input file. The second column lists the subtype label predicted with highest confidence.
  b. Use the *[data-type]-subscope-confidence.txt* file to get confidence scores from subSCOPE for each subtype category.
    ***Note:*** The first row contains the labels for the various subtype categories, and subsequent rows contain confidence values for each input sample. The first column contains sample IDs matching the first column of the input file. Each cell contains a decimal value between [0,1], indicating the confidence score for each (sample, cancer subtype) pair.
    **Pause point:** The runtime for subSCOPE depends on the size of the input features and number of samples, but it does not scale linearly. The data-type is the main factor influencing runtime. Depending upon the data-type selected and the available hardware, it can take up to 3 min – run times are higher for CNVR and METH data-types. Runtime benchmarks for pan-cancer classification across different data-types and sample sizes are summarized in Table 1.
    **CRITICAL:** The accompanying published version of subSCOPE is pre-trained and validated on the NCI GDAN TMP^1^ dataset only, so users should account for potential batch effects when using this method with their own data. Evaluate the method using samples with known labels from each specific dataset, before proceeding with using the predictions. In case of significant batch effects, a potential alternative is to re-train the classifier using each specific dataset.

**Table 1:** subSCOPE runtime benchmarks for various sample sizes and data-types

Data-type	Number of samples in input	Runtime (minutes:seconds)
CNVR	1	3:26
GEXP	1	0:23
METH	1	3:16
MIR	1	0:09
MUTA	1	0:09
CNVR	100	5:00
GEXP	100	0:28
METH	100	4:40
MIR	100	0:13
MUTA	100	0:15

Performance is evaluated on a non-GPU machine with 16 GB memory and a 2.5 GHz quad-core Intel Core i7 processor.

## Expected outcomes

After completion, locate two main .txt files for the aggregated results in the local ‘results’ folder. These files are labelled *[data-type]-subscope-results.txt* and *[data-type]-subscope- confidence.txt*. These result files will provide the highest confidence predicted subtype for each input sample, and granular confidence values for each of the potential subtypes the sample could have been classified as, respectively.

**Figure 5:**
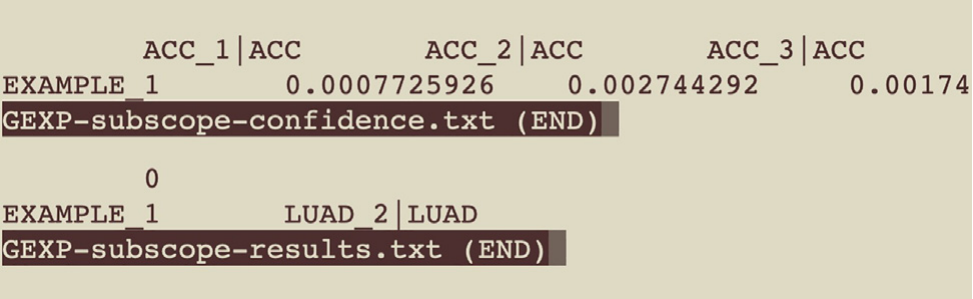
Example output files from subSCOPE for a successful run, data-type GEXP

***Note:*** An example set of output files is shown in Figure 5. Example files are also included as a gzip file in supplemental information as ‘Data S3*’.*

## Limitations

The current version of subSCOPE provides pan-cancer subtype classifications for a new cancer sample based on subtype information across 26 cancer types that have been curated and published^1^ but is not evaluated for other cancer types. The data used for training primarily comes from short-read bulk sequencing approaches and is not evaluated on single-cell data for any data modality. The training and testing data came from the same sources,^1^ limiting the generalizability of the method and the results. For inference and collection of results, subSCOPE requires user expertise in command-line tools. Some of the models themselves, being pan-cancer and ingesting large feature-sets, have a high storage and memory footprint at prediction time – particularly CNVR (24,787 features), which runs in 1–2 min instead of <5 s for the other models on a system with 16 GB RAM and a 2.5 GHz quad-core i7 processor.

## Troubleshooting

### Problem 1

subSCOPE fails to process the input dataset because the matrix has samples as columns and features as rows (step-by-step method details, Step 7).

### Potential solution

In the input matrix, samples should be rows and features should be columns. Transpose the .txt or. tsv input file to follow this format (before you begin, prepare input files).

### Problem 2

subSCOPE fails to process the input dataset because the matrix does not have a header row enumerating the feature names (step-by-step method details, Step 7).

### Potential solution

Please ensure the input file has a first row of feature names defining each column’s values. The feature names must correspond to the GDAN-TMP specific gene IDs. Run the tools/convert.py script provided at the project’s GitHub repository (https://github.com/NCICCGPO/gdan-tmp-models), or follow the tutorial (before you begin, prepare input files).

### Problem 3

subSCOPE fails to process the input dataset because the matrix header does not contain the feature names corresponding to the relevant data-type (step-by-step method details, Step 7).

### Potential solution

Please ensure your input file has the first row where the feature names correspond to the GDAN-TMP specific gene IDs (before you begin, prepare input files).

### Problem 4

Multiple samples are in the input file, but subSCOPE only provides predictions for a single sample (step-by-step method details, Step 9).

### Potential solution

Ensure that the first column of the input file contains sample names. This enables subSCOPE to uniquely process every input sample. If the first column has sample names, verify that each sample name is unique, i.e., no sample name is duplicated. In case of sample name repeats, subSCOPE will default to processing only the first sample entry (before you begin, prepare input files).

### Problem 5

Running ‘docker pull docker.synapse.org/syn29568296/subscope’ results in the error ‘Error response from daemon: pull access denied for docker.synapse.org/syn29568296/subscope, repository does not exist or may require ‘docker login’: denied: requested access to the resource is denied’ (before you begin, Step 5.c).

### Potential solution

Confirm successful login to Docker Desktop on the local machine or remote system where Docker is intended to run. Rerun `docker pull` after you have verified a successful Docker login.

### Problem 6

The predictions on the RNA-seq or miRNA-seq dataset are noisy (expected outcomes).

### Potential solution

Ensure that expression data has been quantile rescaled. If not, then run script `run_transform.sh`, as shown in the tutorial at https://github.com/NCICCGPO/gdan-tmp-models (before you begin, prepare input files).

## Resource availability

### Lead contact

Further information and requests for resources and reagents should be directed to and will be fulfilled by the lead contact, Dr. Steven J.M. Jones (sjones@bcgsc.ca).

### Technical contact

Technical questions on executing this protocol should be directed to and will be answered by the technical contact, Dr. Jasleen K. Grewal (jgrewal@nvidia.com).

### Materials availability

This study did not generate new unique reagents or materials.

### Data and code availability

• All original code has been deposited at GitHub and is publicly available as of the date of publication at https://github.com/NCICCGPO/gdan-tmp-models/tree/main/subscope.
• Any additional information required to reanalyze the data reported in this paper is available from the lead contact upon reasonable request.
• Supplementary data has been deposited to Mendeley Data, https://doi.org/10.17632/vd6tct9xwr.1.

## Consortia

The Cancer Genome Atlas Analysis Network: Theo A. Knijnenburg, Mauro A. A. Castro, Vinicius S. Chagas, Victor H. Apolonio, Verena Friedl, Joshua M. Stuart, Vladislav Uzunangelov, Christopher K. Wong, Jesper B. Andersen, Andrew D. Cherniack, Galen F Gao, Gad Getz, Stephanie H. Hoyt, Whijae Roh, Lindsay Westlake, Christopher C Benz, Jasleen K. Grewal, Steven J.M. Jones, A. Gordon Robertson, Samantha J. Caesar-Johnson, John A. Demchok, Ina Felau, Anab Kemal, Roy Tarnuzzer, Zhining Wang, Liming Yang, Jean C. Zenklusen, Rehan Akbani, Bradley M. Broom, Zhenlin Ju, Andre Schultz, Akinyemi I. Ojesina, Katherine A. Hoadley, Avantika Lal, Daniele Ramazzotti, Chen Wang, Alexander J. Lazar, Lewis R. Roberts, Taek-Kyun Kim, Ilya Shmulevich, Bahar Tercan, Paulos Charonyktakis, Vincenzo Lagani, Ioannis Tsamardinos, Esther Drill, Ronglai Shen, Martin L. Ferguson, Kami E Chiotti, Kyle Ellrott, Brian J. Karlberg, Jordan A. Lee, Eve Lowenstein, Adam Struck, Paul T. Spellman, Christina Yau, Toshinori Hinoue, Peter W. Laird.

## Acknowledgments

We would like to thank Canada’s Michael Smith Genome Sciences Center and the 10.13039/100000054National Cancer Institute for their support. Discussions and feedback from the GDAN TMP group were extremely helpful in improving subSCOPE. This work was supported, in part, by the 10.13039/100000002U.S. National Institutes of Health grant 5U24CA210952-05 to S.J.M.J., U24CA264029 to A.D.C., and U24CA264023 to P.W.L.

## Author contributions

S.J.M.J. and J.K.G. conceptualized, implemented, and evaluated the initial method. M.A.A.C., K.E., J.A.L., Brian J. Karlberg, J.K.G., C.Y., B.T., and A.G.R. jointly undertook validation, formal analysis, and data curation. S.J.M.J., M.A.A.C., K.E., J.A.L., Brian J. Karlberg, J.K.G., C.K.W., and B.T. created and evaluated the final deliverables. Writing and editing were done by J.K.G., A.G.R., P.W.L., B.T., Brian J. Karlberg, and S.J.M.J. Project supervision was provided by S.J.M.J., P.W.L., J.C.Z., C.C.B., and A.D.C. Project administration and funding were overseen and secured by S.J.M.J., P.W.L., J.C.Z., and A.D.C.

## Declaration of interests

A.D.C. receives research support from Bayer and consults for KaryoVerse.

## Supplemental information

Document S1. Training guide and summary

Data S1. Supplemental gzip file with 5 feature lists, each corresponding to each DATA TYPE, related to step 1 (a) and "before you begin" step 3 (a)

Data S2. Example input file for subSCOPE, related to step 2 and "before you begin" step 3 This is a computer generated example for purposes of demonstration only.

Data S3. Example output files for subSCOPE, related to "expected outcomes"
