# Supplementary material for: Protocol for obtaining cancer type and subtype predictions using subSCOPE
Source: STAR Protoc. 2025 Apr 10;6(2):103705. doi: 10.1016/j.xpro.2025.103705 (PMC12013717; doi:10.1016/j.xpro.2025.103705)
Supplement: Document S1. Training guide and summary [file mmc1.pdf]

The pre-trained subSCOPE model described in the main protocol is trained using Model Training Data noted in **Key Resources Table** and following the two general recipes described here**.**

subSCOPE training (optional)

Note: Training each of the 25 models in subSCOPE to convergence takes 8-10 hours per model, assuming training on a single NVIDIA V100 GPU with 512 GB system memory.

This section describes the general training process for subSCOPE as optional background information. Adapt these guidelines for your machine learning setup to train the subSCOPE method.

- Organize samples by data-type (CNVR, GEXP, METH, MIR, MUTA) following the file format descibed in “Before you being > Prepare input files”
- Prepare training data for each data-type into training and testing sets with a 5-fold cross-validation approach.
- Use your preferred machine learning implementation approach to train a feed-forward neural network with a single hidden layer, for each data-type and each cross-validation split.
    - In each ‘run’, train across all cancer types and subtypes using the following routine for the training folds:
        - Initialize the network with random weights (random seed 1234) and pre-train for 20 epochs to classify all cancer types. Save this model.
        - Next, fully train this model with the objective of classifying all subtypes across all cancer types using the same hyperparameters and same train/test data splits.
          Note: Replace the output layer to accommodate all subtypes instead of all cancer types for this change.
    - For each network, keep a randomly sampled 10% of training samples as validation samples.
        - Compare training loss with validation loss on these samples. Stop training automatically if the two losses show a divergent trend for more than three consecutive epochs.
          Note: Alternatively, implement this routine with early-stopping.
        - Set data-type specific maximum training epochs. Train the network for this maximum number of epochs unless it stops earlier with early-stopping.
- Review Table 1 to understand the network architecture details, hyperparameters, and number of maximum training epochs used to train the provided version of subSCOPE.
  Note: Set the following shared parameters: Batch normalization layer before the output layer, weighted loss based on size of each classification category (‘*class_weights*’), and multiclass focal loss with parameters alpha = 5 * *class_weights*, gamma = 4.

Table S1. Training parameters for subSCOPE models, as determined by hyperparameter optimization.

Data-type	Neural Network Architecture (input x hidden x output)	L1 regularizer	L2 regularizer	Hidden layer activation	Dropout	Learning rate	Maximum training epochs
GEXP	20530 x 500 x 106	0.01	0.1	Tanh	0.75	0.001	1000
METH	35743 x 5000 x 104	0.001	0.01	Tanh	0.0	0.001	1000
MUTA	4279 x 150 x 104	0.0	0.001	LeakyReLU	0.1	0.001	600
CNVR	74363 x 500 x 104	0.001	0.0	LeakyReLU	0.75	0.001	1000
MIR	742 x 500 x 104	0.0	0.1	Tanh	0.75	0.001	1000

subSCOPE hyperparameter optimization (optional)

Note: Evaluating the various hyperparameter settings robustly takes 24-72 hours per data-type (hyperparameter optimization on four NVIDIA V100 GPUs, each with 512 GB system memory)

Review this sub-section to independently determine an optimal set of hyperparameters for each model. Alternatively, use the previously determined best parameters as described in **Table 1**.

Selection of appropriate parameter values for each data-type specific model:

- Evaluate convergence and cross-validation performance for learning rate values of [0.1, 0.01, 0.001].
- Evaluate convergence and cross-validation performance for dropout values of [0.0, 0.1, 0.25, 0.5, 0.75, 0.9].
  Note: Dropout performs random masking of input features and has been known to improve generalizability and prevent overfitting in neural network models.
- Evaluate the effect of L2 regularization on performance for each data-type, exploring L2 values of [0.1, 0.01, 0.001, 0.0].
- Evaluate the effect of L1 regularization using the best determined learning rate and L2 parameters, exploring L1 values of [0.1, 0.01, 0.001, 0.0].
- Next evaluate the effect of the hidden layer size across the options [50, 150, 500, 1000, 5000].
- Increase the hidden layer size incrementally starting from 100 only if the 150 nodes indicate a notable increase in performance (1 point improvement in F1-score).
- Evaluate the maximum number of epochs starting at 200. If the training consistently terminates at 200 with the validation loss curve trending towards convergence, the epochs are iteratively increased in steps of 200 until a maximum of 1000 epochs.
  Note: While additional epoch steps were not evaluated in the interest of training time and efficiency, converge was achieved earlier than 1000 epochs in all trained models included here.

Process to aggregate various trained models into an ensemble of data-type specific models:

- Train five models for each data-type, one for each different cross-validation folds.
- For each data-type, follow these sub-steps to combine the five models into an ensemble of models.
    - For each sample at prediction time, collect the predictions from each member of the ensemble with accompanying confidence scores.
    - Calculate an average prediction confidence value for each prediction category across the 5 models.

Alternatives: For the published pre-trained subSCOPE model, additional criteria were evaluated when determining the best modeling approach, including evaluation of pan-cancer versus cancer-specific classifiers, other architectural designs, other hyperparameter choices and minor algorithmic optimizations. These are listed here briefly for the interest of the reader:

- Compare performance of pan-cancer versus cancer-specific classifiers (using GEXP data)
- Evaluate benefit of feature selection based on variance thresholding in cancer-specific classifier training (using GEXP data)
- Evaluate feature importance evaluation methods - DeepLift[1] (tractable for large feature sets) on the pan-cancer networks, and the SHAP[2] method (tractable for smaller feature sets) on a BRCA-subtypes classifier (GEXP data). SHAP and LIME[3] scaled poorly to the large pan-cancer models.
- Evaluate effect of class-balancing in loss calculation for pan-cancer classifier training (using GEXP data)
- Compare focal loss[4] and cross-entropy loss[5] for multi-class classification (pan-cancer classifier, GEXP data).
- Compare activation functions and their impact on cancer-specific classifiers (using GEXP data), namely tanh, selu, relu[6].

Supplemental References

1. Shrikumar, A., Greenside, P. and Kundaje, A., 2017. Learning important features through propagating activation differences. In *International conference on machine learning* (pp. 3145-3153). PMLR.
2. Lundberg, S.M. and Lee, S.I., 2017. A unified approach to interpreting model predictions. *Advances in neural information processing systems*, *30*.
3. Ribeiro, M.T., Singh, S. and Guestrin, C., 2016, August. " Why should I trust you?" Explaining the predictions of any classifier. In *Proceedings of the 22nd ACM SIGKDD international conference on knowledge discovery and data mining* (pp. 1135-1144).
4. Lin, T.Y., Goyal, P., Girshick, R., He, K. and Dollár, P., 2017. Focal loss for dense object detection. In *Proceedings of the IEEE international conference on computer vision* (pp. 2980-2988).
5. Murphy, Kevin P. *Machine learning: a probabilistic perspective*. MIT press, 2012.

6. Klambauer, G., Unterthiner, T., Mayr, A. and Hochreiter, S., 2017. Self-normalizing neural networks. *Advances in neural information processing systems*, *30*.
